# High Salt Intake Damages the Heart through Activation of Cardiac (Pro) Renin Receptors Even at an Early Stage of Hypertension

**DOI:** 10.1371/journal.pone.0120453

**Published:** 2015-03-23

**Authors:** Yuka Hayakawa, Takuma Aoyama, Chiharu Yokoyama, Chihiro Okamoto, Hisaaki Komaki, Shingo Minatoguchi, Masamitsu Iwasa, Yoshihisa Yamada, Itta Kawamura, Masanori Kawasaki, Kazuhiko Nishigaki, Atsushi Mikami, Fumiaki Suzuki, Shinya Minatoguchi

**Affiliations:** 1 Department of Cardiology, Gifu University Graduate School of Medicine, Yanagido, Gifu, Japan; 2 Department of Life Science, Gifu University, Yanagido, Gifu, Japan; Universidade Federal do Rio de Janeiro, BRAZIL

## Abstract

**Objective:**

It has not yet been fully elucidated whether cardiac tissue levels of prorenin, renin and (P)RR are activated in hypertension with a high salt intake. We hypothesized that a high salt intake activates the cardiac tissue renin angiotensin system and prorenin-(pro)renin receptor system, and damages the heart at an early stage of hypertension.

**Methods:**

Wistar Kyoto rats (WKY) and spontaneously hypertensive rats (SHR) received regular (normal-salt diet, 0.9%) and high-salt (8.9%) chow for 6 weeks from 6 to 12 weeks of age. The systolic blood pressure, plasma renin activity (PRA) and plasma angiotensin II concentration were measured, and the protein expressions of prorenin, (pro)renin receptor, angiotensinogen, angiotensin II AT1 receptor, ERK1/2, TGF-β, p38MAPK and HSP27 in the myocardium were investigated. The cardiac function was assessed by echocardiography, and histological analysis of the myocardium was performed.

**Results:**

The high-salt diet significantly increased the systolic blood pressure, and significantly reduced the PRA and plasma angiotensin II concentration both in the WKYs and SHRs. Cardiac expressions of prorenin, renin, (P)RR, angiotensinogen, angiotensin II AT1 receptor, phosphorylated (p)-ERK1/2, p-p38MAPK, TGF-β and p-HSP27 were significantly increased by the high salt diet both in the WKYs and SHRs. The high-salt diet significantly increased the interventricular septum thickness and cardiomyocyte size, and accelerated cardiac interstitial and perivascular fibrosis both in the WKYs and SHRs. On the other hand, dilatation of left ventricular end-diastolic dimension and impairment of left ventricular fractional shortening was shown only in salt loaded SHRs.

**Conclusion:**

The high-salt diet markedly accelerated cardiac damage through the stimulation of cardiac (P)RR and angiotensin II AT1 receptor by increasing tissue prorenin, renin and angiotensinogen and the activation of ERK1/2, TGF-β, p38MAPK and HSP27 under higher blood pressure.

## Introduction

The renin angiotensin (RA) system is a major regulator of the blood pressure [1] and has been considered to be involved in the development and progression of hypertensive heart disease [2]. Renin and its precursor prorenin are usually produced from granular cells of the juxtaglomerular apparatus in the terminal afferent arteriole in the kidney [3]. Recently, the renal tubular segment, including the collecting duct, has also been reported to be a source of prorenin [4]. Renin and prorenin bind to the (pro)renin receptor ((P)RR). Binding of prorenin to the (P)RR has been reported to be involved in the development of diabetic nephropathy [5]. Additionally, it was reported that the blockade of prorenin reduced the left ventricular mass and improved the left ventricular function in spontaneously hypertensive rats (SHRs) with cardiovascular damage due to excess salt, suggesting the involvement of prorenin in cardiac damage [6].

Generally, plasma renin activity (PRA) increases with a decrease in salt intake and decreases with an increase in salt intake [7, 8]. However, the tissue RA system sometimes behaves oppositely to plasma levels of renin. The tissue RA system is activated in diabetes, although plasma renin levels are paradoxically low [9, 10]. It has already been reported that salt overload induces cardiac hypertrophy and interstitial fibrosis via activation of cardiac angiotensin II type 1 receptor due to a blood pressure-independent mechanism in Wistar rats [11, 12]. However, it has still not been fully elucidated whether cardiac tissue levels of prorenin, renin, (P)RR, angiotensin II and angiotensin II AT1 receptor are activated at an early stage of hypertension with a high salt intake. We hypothesized that a high salt intake causes an increase in tissue levels of prorenin, renin, (P)RR, angiotensin II and angiotensin II AT1 receptor, and damages the heart at an early stage of hypertension. Therefore, in the present study, we investigated the relationship between the plasma RA system and cardiac tissue RA system, the roles of cardiac (P)RR, angiotensin II AT1 receptor and their signal transduction in the development of cardiac damage in young WKYs and SHRs with a high salt intake.

## Materials and Methods

### Experimental animals and ethics statement

Six-week-old male spontaneously hypertensive rats/ Izm (SHRs) and Wistar Kyoto rats/ Izm (WKYs), maintained in specific pathogen-free conditions at Japan SLC Inc. (Shizuoka, Japan), were purchased from Chubu Kagaku Sizai Co., Ltd. (Nagoya, Japan). Rats were housed solely or in pairs in a single cages 26 cm(W) × 42 (D) × 18 (H) in size, and maintained in temperature (23 ± 2°C)- and humidity (65 ± 5%)-controlled animal rooms with a 12-h light and 12-h dark cycle. All rats were allowed free access to water and diet. The staff in the animal house took care of animals every day, and rats were given diet and water. We checked the conditions of animals and measured blood pressure once a week. All animal experiments were carried out in strict accordance with the recommendations of the Standards Relating to the Care and Management of Laboratory Animals and Relief of Pain (2006) published by the Japanese Ministry of the Environment, and were also handled in accordance with the Guide for the Care and Use of Laboratory Animals, published by the US National Institutes of Health (NIH Publication, 8th Edition, 2011). The study protocol was approved by the Committee for Animal Research and Welfare of Gifu University, Gifu, Japan (Permit Number: 24-9). At the end of the experiment, echocardiography was performed under sodium pentobarbital light anesthesia (Tokyo Chemical Industry Co., Ltd., Tokyo, Japan) to take a rest position. After echocardiography, the catheter was cannulated into the femoral vein to take blood samples under diethyl ether anesthesia (Wako Pure Chemical Industries, Ltd., Osaka, Japan) to minimize the pain, and rats were euthanized by exsanguinations until righting reflex was lost. After sacrifice, organs were removed from all rats. All efforts were made to minimize suffering. During the course of this study, none of the rats died.

### Protocol

**Fig 1:**
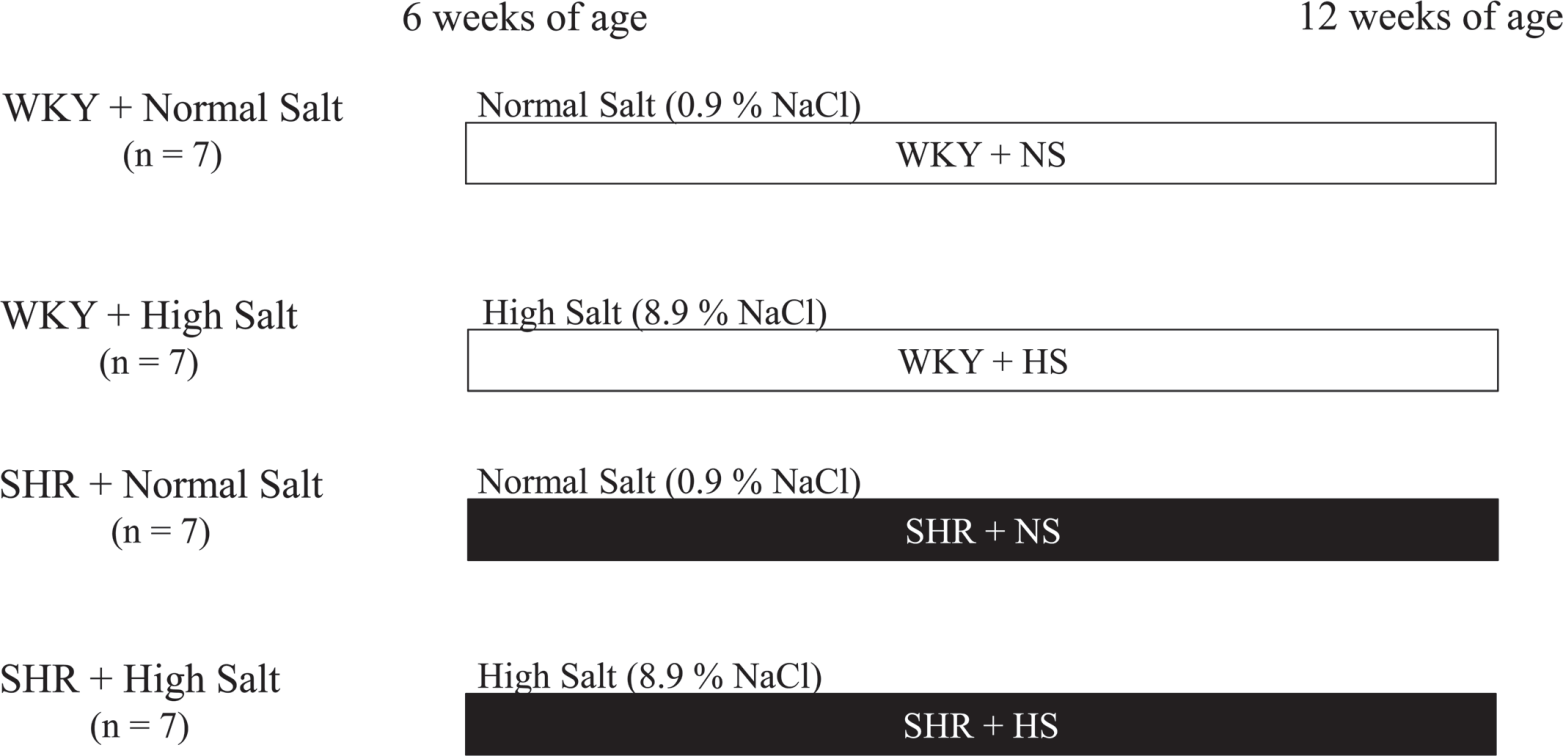
Protocol of the experiment.

As shown in Fig. 1, SHRs and WKYs at 6 weeks old received regular rat chow (normal-salt diet, 0.9% NaCl, CE-2; CLEA Japan, Inc., Tokyo, Japan) or high-salt chow (CE-2 + 8% NaCl: 8.9% NaCl; CLEA Japan, Inc.) for 6 weeks from 6 to 12 weeks of age (n = 7, respectively). Rats were randomly assigned to either normal salt diet group or high salt diet group. The study groups were WKY + normal-salt (WKY+NS), WKY + high-salt (WKY+HS), SHR + normal-salt (SHR+NS) and SHR + high-salt (SHR+HS) groups.

### Measurement of blood pressure

Systolic blood pressure was measured once a week for 6 weeks from 6 to 12 weeks of age by the tail-cuff method (BP98-A; Softron Co., Ltd., Tokyo, Japan) in all rats.

### Echocardiography

Echocardiography (Vevo 770; Visualsonics, Toronto, Canada, equipped with a 45-MHz imaging transducer) was performed at 12 weeks of age, and left ventricular fractional shortening (FS), left ventricular end-diastolic dimension (LVDd) and interventricular septal (IVS) thickness were obtained.

### Measurement of plasma renin activity (PRA) and plasma angiotensin II concentration

At the end of the experiment, all rats were lightly anesthetized with diethyl ether for relief of pain, and the catheter was cannulated into the femoral vein to take blood samples (∼8 mL each) for the measurement of PRA and plasma angiotensin II concentration. PRA and plasma angiotensin II concentration were measured using the RIA2 method (SRL, Inc., Tokyo, Japan).

### Western blot analysis

At the end of the experiment at 12 weeks of age, the heart was excised. Western blot analysis was carried out using homogenates from the heart tissues. Proteins were separated and transferred to membranes using standard protocols, after which they were probed with antibodies against prorenin and renin (1:100; Santa Cruz Biotechnology, Inc., Dallas, Texas, USA), (pro)renin receptors (1:100; Santa Cruz Biotechnology, Inc.), angiotensinogen (1:200; Phoenix Pharmaceuticals, Inc., Burlingame, CA, USA), angiotensin II AT1 receptor (1:200; Enzo Life Sciences, Inc., Farmingdale, NY, USA), extracellular signal-related kinases (ERK)1/2 (1:100; Cell Signaling Technology, Inc., Danvers, MA, USA), phosphorylated (p)-ERK1/2 (1:100; Cell Signaling Technology, Inc.), transforming growth factor (TGF)- β1 (1:200; Santa Cruz Biotechnology, Inc.), p38 mitogen-activated protein kinase (MAPK) (1:100; Cell Signaling Technology, Inc.), p-p38MAPK (1:100; Cell Signaling Technology, Inc.), heat shock protein (HSP)27 (1:100; Santa Cruz Biotechnology, Inc.) and p-HSP27 (1:100; Santa Cruz Biotechnology, Inc.). The blots were visualized by means of chemiluminescence (ECL; GE Healthcare UK Ltd., Amersham Place, Buckinghamshire, England), and the signals were quantified by densitometry. GAPDH (analyzed with an antibody from Cell Signaling Technology, Inc.) served as the loading control.

### Pathology

At the end of the experiment, the heart was excised, and the left ventricle was weighed and sectioned into two transverse slices parallel to the atrioventricular ring. Each slice was then fixed in 10% buffered formalin for 4 h, embedded in paraffin, and cut into 4-μm-thick sections with a microtome. These sections were stained with hematoxylin-eosin and Masson-Trichrome. The above stained preparations were observed by light microscopy.

From the sections stained with Masson-Trichrome, the ratio of the myocardial interstitial fibrosis area divided by the total myocardium area was obtained. From the sections stained with hematoxylin-eosin, the diameter of cardiomyocytes was measured. The measurements were taken along the short axis of the cardiomyocytes at the level of nucleus. These were performed by 2 persons blinded to treatment.

### Statistical analysis

All values are presented as the means ± SEM. The normality of distributions was tested using the Kolmogorov-Smirnov test. Differences in parameters at 12 weeks of age among the four groups were assessed by one way analysis of variance (ANOVA) followed by Fisher’s method for post hoc comparisons. Two-way ANOVA was used to detect differences in blood pressure at 6week to 12 week among four groups followed by Fisher’s method for post hoc comparisons. Values of p < 0.05 were considered significant. Statistical analyses were performed using Stat View version 5.0 (SAS Institute Inc., Cary, NC, USA).

## Results

### Blood pressure

All date were normally distributed.

**Fig 2:**
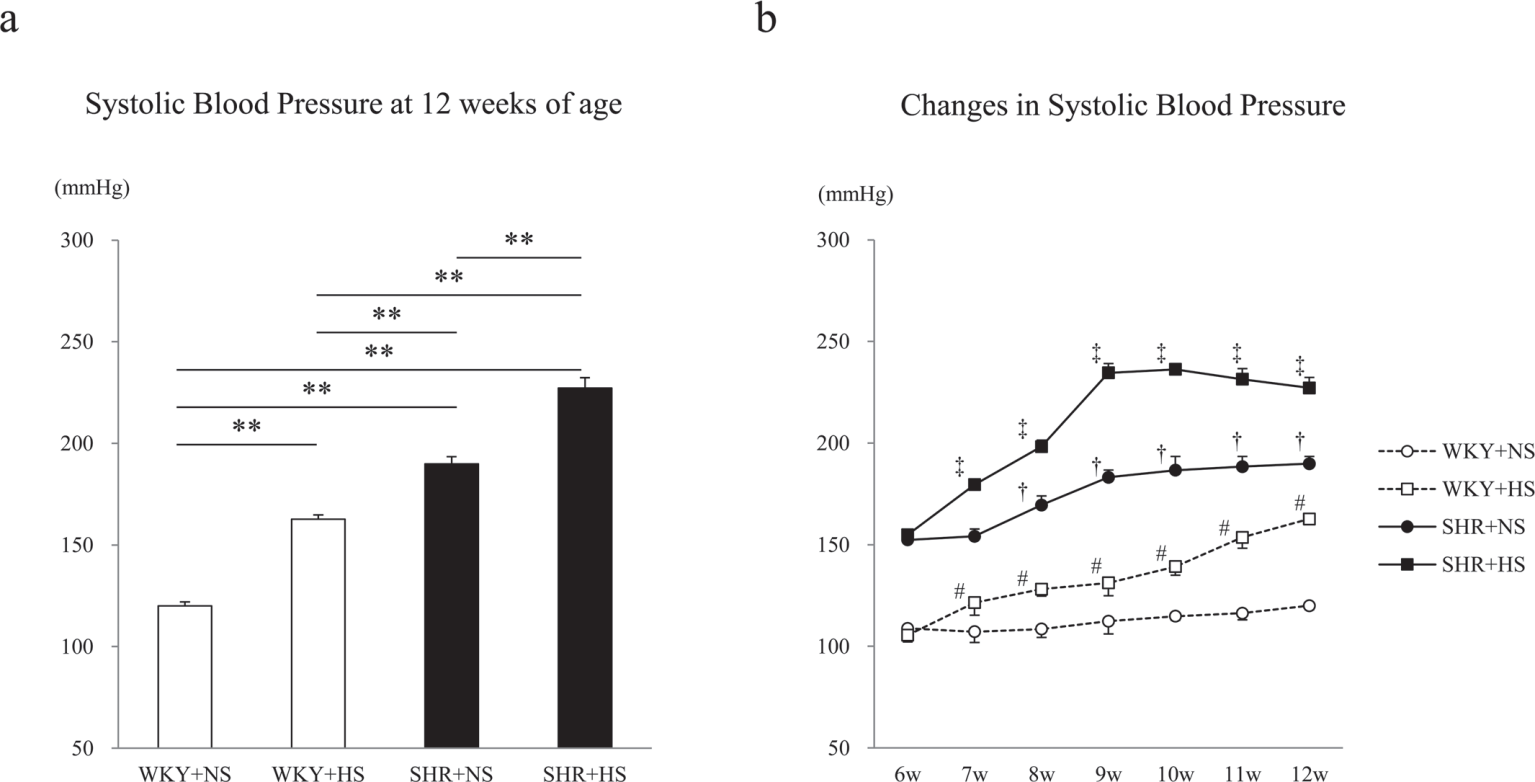
Changes in the systolic blood pressure caused by a high salt intake in the WKY and SHR groups. (a) Systolic blood pressure at 12 weeks of age in the WKY + normal-salt group (WKY+NS), WKY + high-salt group (WKY+HS), SHR + normal-salt group (SHR+NS) and SHR + high-salt group (SHR+HS). Data represent the means ± SEM (n = 7). **: p < 0.01, *: p < 0.05. (b) Time-course changes in systolic blood pressure in response to a high salt intake in the WKY and SHR groups. Data represent the means ± SEM (n = 7). #: p < 0.05 vs. 6 weeks of age of WKY + HS, †: p < 0.05 vs. 6 weeks of age of SHR + NS, ‡: p < 0.05 vs. 6 weeks of age of SHR + HS.

As shown in Fig. 2A, at 12 weeks of age, the systolic blood pressure was significantly higher in the SHR+NS group (190 ± 3.57 mmHg) than in the WKY+NS group (120 ± 1.94 mmHg) (p < 0.001), and the WKY+HS group (163 ± 2.20 mmHg) and SHR+HS group (227 ± 5.12 mmHg) showed a higher systolic blood pressure than the WKY+NS group (p < 0.001) and SHR+NS group (p < 0.001), respectively. As shown in Fig. 2B, the high-salt diet gradually and significantly increased the systolic blood pressure from 6 to 12 weeks of age both in the WKY and SHR groups as compared to those fed the normal-salt diet. The SHR+HS group showed a gradual but significant increase in the systolic blood pressure from 6 to 12 weeks of age.

### Heart weight, lung weight, body weight, heart weight/ body weight ratio and lung weight/ body weight ratio

**Fig 3:**
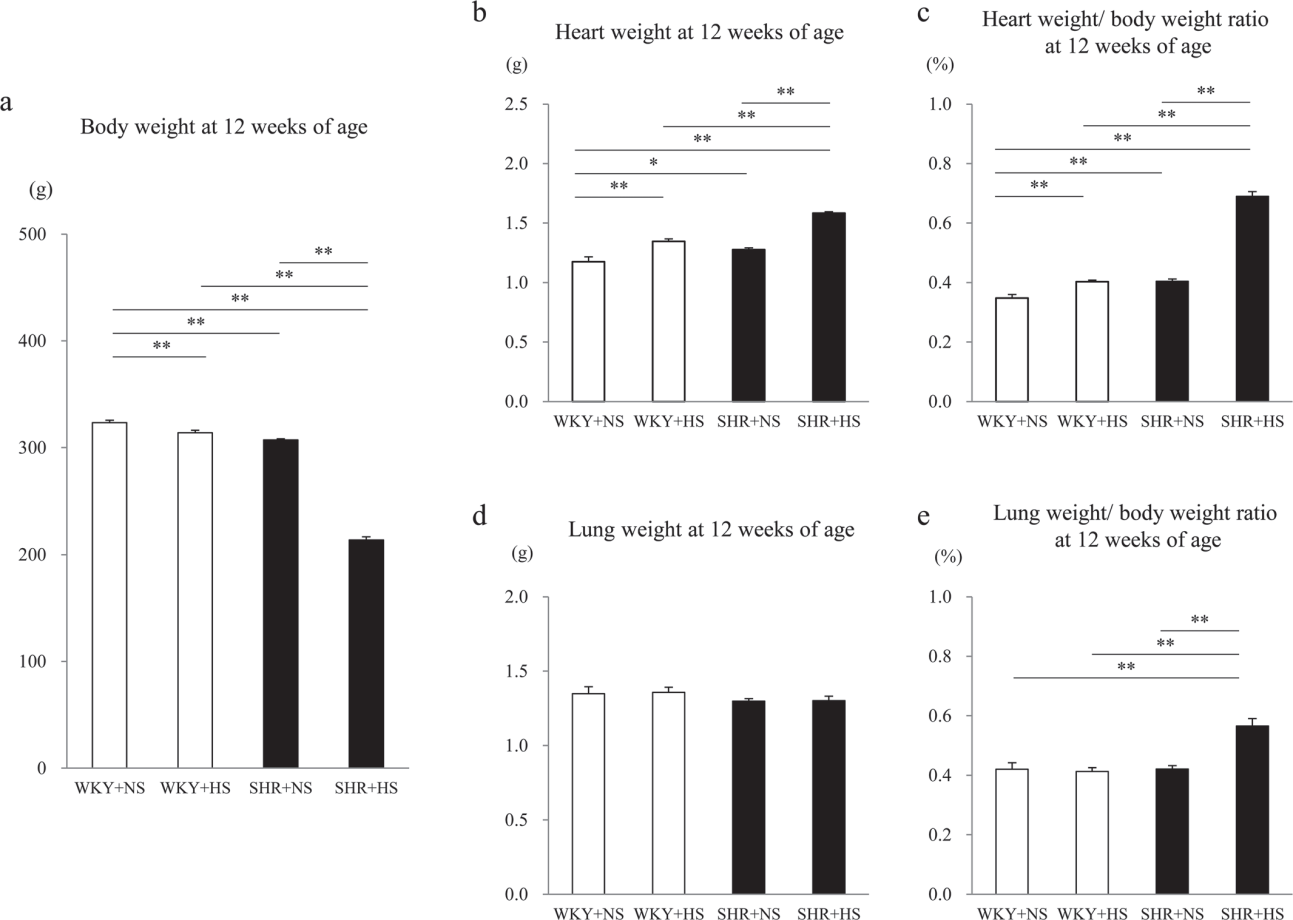
Heart weight, lung weight, body weight, heart weight/ body weight ratio and lung weight/ body weight ratio. (a) Body weight, (b) Heart weight, (c) Heart weight/ body weight ratio, (d) Lung weight and (e) Lung weight/ body weight ratio at 12 weeks of age in the WKY + normal-salt group (WKY+NS), WKY + high-salt group (WKY+HS), SHR + normal-salt group (SHR+NS) and SHR + high-salt group (SHR+HS). Data represent the means ± SEM (n = 7). **: p < 0.01, *: p < 0.05.

As shown in Fig. 3B, at 12 weeks of age, the heart weight was significantly heavier in the WKY+HS group (1.35 ± 0.02 g) and SHR+HS group (1.58 ± 0.01 g) than in the WKY+NS group (1.18 ± 0.04 g) (p < 0.001) and SHR+NS group (1.28 ± 0.01 g) (p < 0.001), respectively. The heart weight was significantly heavier in the SHR+HS group than in the other 3 groups. On the contrary, the body weight was significantly lighter in the SHR+HS group (214 ± 3 g) than in the WKY+NS group (323 ± 2 g) (p < 0.001), WKY+HS group (314 ± 2 g) (p < 0.001) and SHR+NS group (307 ± 1 g) (p < 0.001). The heart weight/ body weight ratio was significantly higher in the WKY+HS group (0.40 ± 0.01%) and SHR+HS group (0.69 ± 0.02%) than in the WKY+NS group (0.35 ± 0.01%) (p = 0.004) and SHR+NS group (0.40 ± 0.01%) (p < 0.001), respectively. The heart weight/ body weight ratio was significantly higher in the SHR+HS group than in the other 3 groups. The lung weight was not different among groups (The lung weights were 1.35 ± 0.05, 1.35 ± 0.03, 1.30 ± 0.02 and 1.30 ± 0.03 g in the WKY+NS, WKY+HS, SHR+NS and SHR+HS groups, respectively.). However, the ratios of lung weight/ body weight were 0.42 ± 0.02, 0.41 ± 0.01, 0.42 ± 0.01 and 0.57 ± 0.02% in the WKY+NS, WKY+HS, SHR+NS and SHR+HS groups, respectively. The lung weight/ body weight ratio was significantly higher in the SHR+HS group than in the other 3 groups.

### Echocardiography

**Fig 4:**
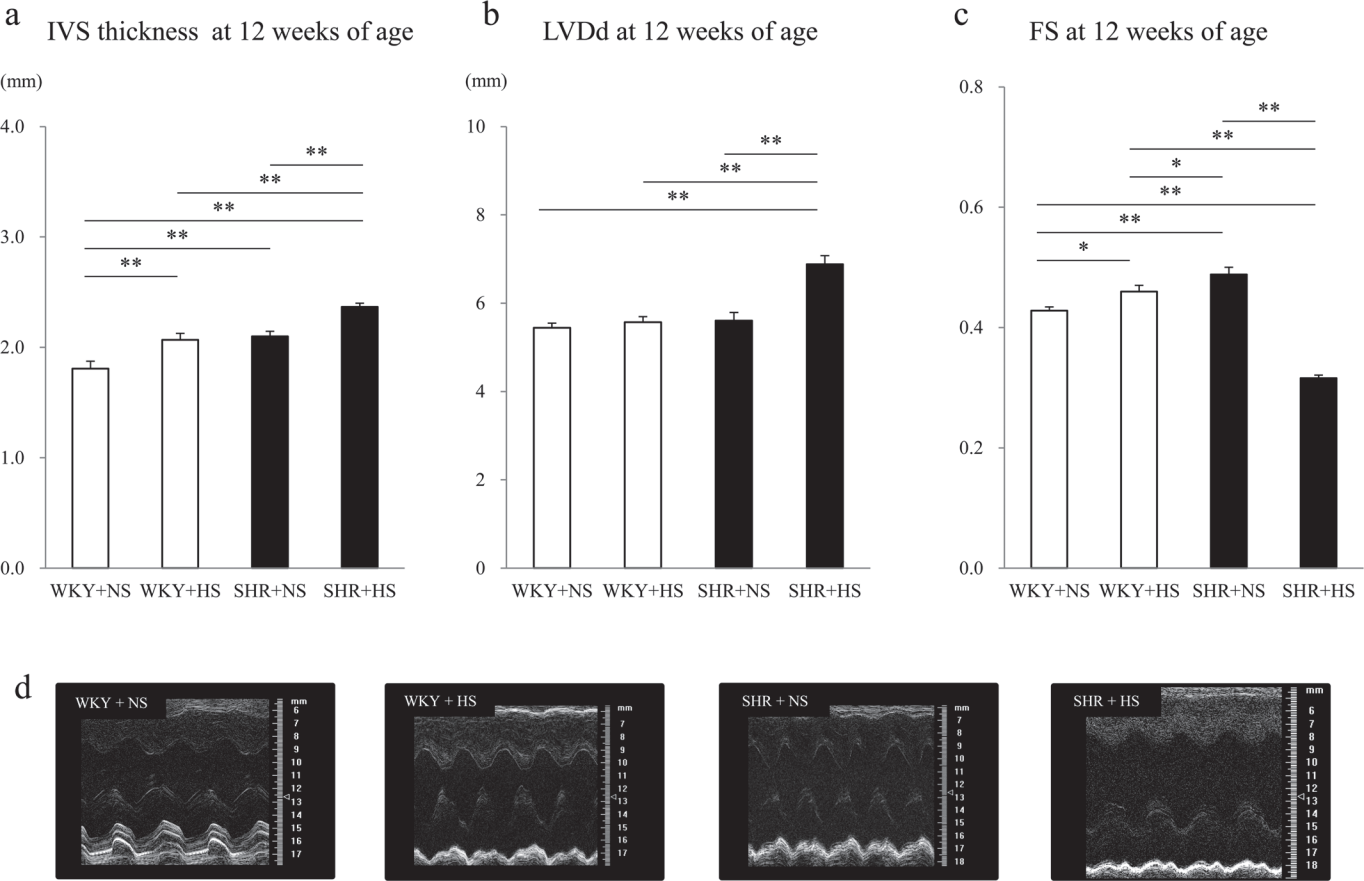
Echocardiographic findings. (a) Interventricular septum (IVS) thickness, (b) Left ventricular diastolic dimension (LVDd) and (c) Fractional shortening (FS) at 12 weeks of age in the WKY + normal-salt group (WKY+NS), WKY + high-salt group (WKY+HS), SHR + normal-salt group (SHR+NS) and SHR + high-salt group (SHR+HS). Data represent the means ± SEM (n = 7). **: p < 0.01, *: p < 0.05. (d) Representative M-mode echo cardiograms at left ventricular levels.

As shown in Fig. 4, the interventricular septum (IVS) thickness, an indicator of left ventricular hypertrophy, was significantly greater in the WKY+HS group (2.07 ± 0.06 mm) than in the WKY+NS group (1.81 ± 0.07 mm) (p = 0.003), and greater in the SHR+HS group (2.37 ± 0.03 mm) than in the SHR+NS group (2.10 ± 0.05 mm) (p = 0.002). The IVS was significantly thicker in the SHR+HS group than in the other 3 groups. The left ventricular end-diastolic dimension (LVDd) was significantly greater in the SHR+HS group (6.88 ± 0.19 mm), and fractional shortening (FS) was significantly smaller in the SHR+HS group (0.32 ± 0.01) than in the other 3 groups. (LVDd were 5.45 ± 0.10, 5.57 ± 0.13 and 5.61 ± 0.19 mm in the WKY+NS, WKY+HS and SHR+NS groups, respectively. FS were 0.43 ± 0.01, 0.46 ± 0.01 and 0.49 ± 0.01 in the WKY+NS, WKY+HS and SHR+NS groups, respectively.)

### Plasma renin activity (PRA) and plasma angiotensin II concentration

**Fig 5:**
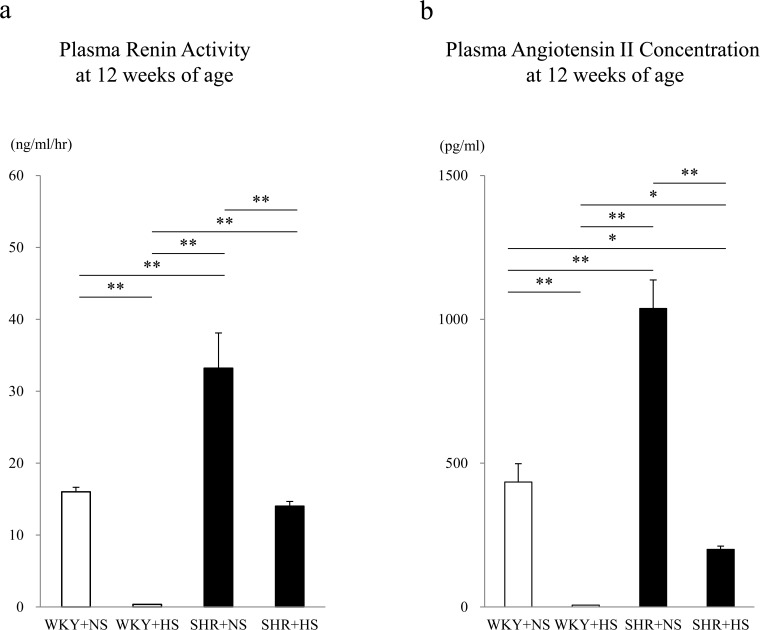
Plasma renin activity and plasma angiotensin II concentration. (a) Plasma renin activity and (b) Plasma angiotensin II concentration in the WKY + normal-salt group (WKY+NS), WKY + high-salt group (WKY+HS), SHR + normal-salt group (SHR+NS) and SHR + high-salt group (SHR+HS). Data represent the means ± SEM (n = 7). **: p < 0.01, *: p < 0.05.

PRA were 16.00 ± 0.63, 0.34 ± 0.04, 33.20 ± 4.90 and 14.00 ± 0.67 ng/mL/hr in the WKY+NS, WKY+HS, SHR+NS and SHR+HS groups, respectively. Plasma renin activity (PRA) was significantly higher in the SHR + NS group than in the WKY + NS group (p < 0.001) (Fig. 5A). The high-salt diet significantly decreased PRA both in the WKYs (p = 0.001) and SHRs (p < 0.001). Plasma angiotensin II concentrations were 434 ± 64, 19.6 ± 0.9, 1,038 ± 99 and 200 ± 11 pg/mL in the WKY+NS, WKY+HS, SHR+NS and SHR+HS groups, respectively. The plasma angiotensin II concentration was significantly higher in the SHR + NS group than in the WKY + NS group (p < 0.001). The high-salt diet significantly decreased the plasma angiotensin II concentration both in the WKYs (p < 0.001) and SHRs (p < 0.001) (Fig. 5-B).

### Expressions of cardiac tissue prorenin, renin, and (P)RR

**Fig 6:**
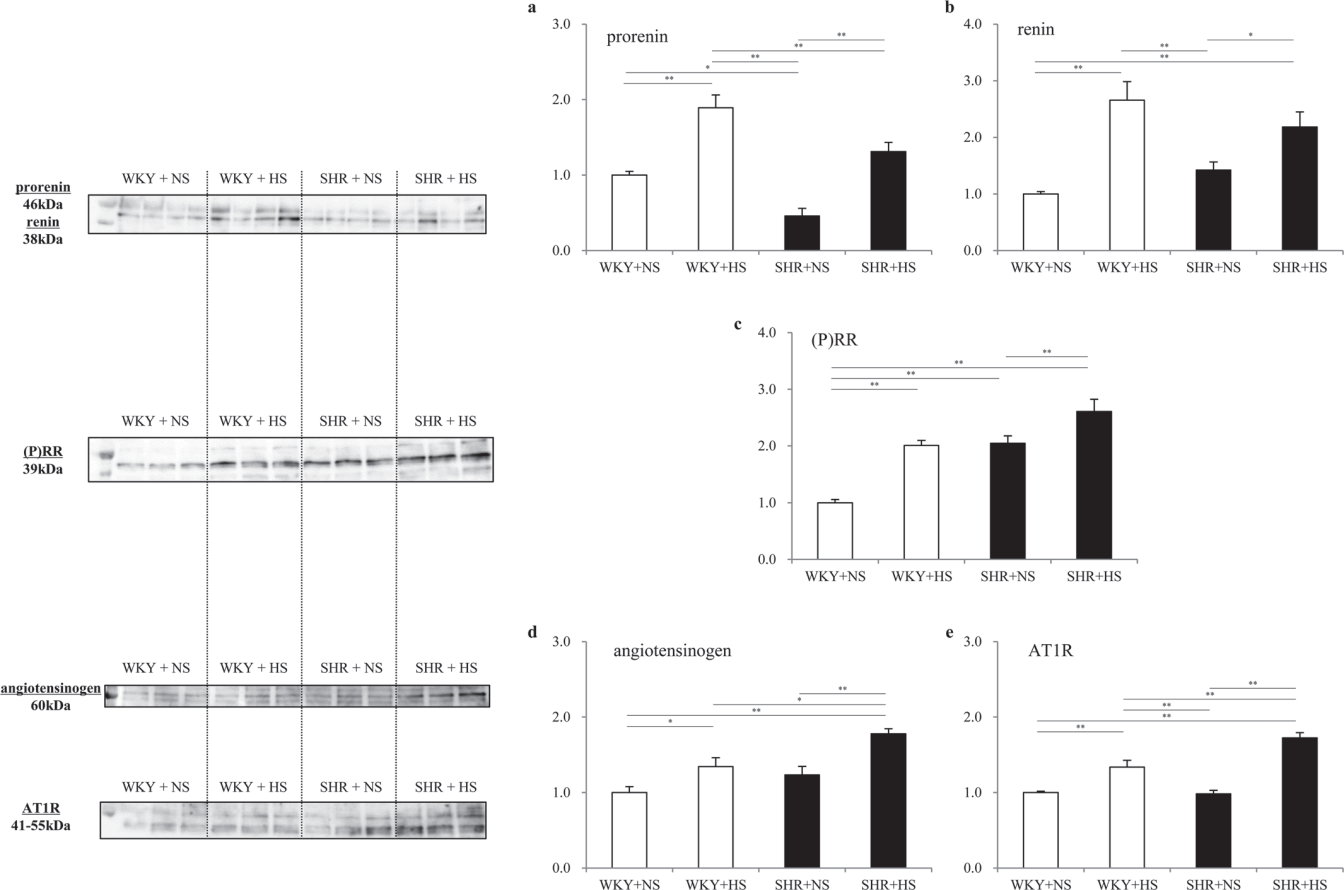
Expression of cardiac tissue prorenin, renin, (pro)renin receptor, angiotensinogen and angiotensin II AT1 receptor. Expressions of cardiac tissue (a) prorenin, (b) renin, (c) (pro)renin receptor, (d) angiotensinogen and (e) angiotensin AT1 receptor in the WKY + normal-salt group (WKY+NS), WKY + high-salt group (WKY+HS), SHR + normal-salt group (SHR+NS) and SHR + high-salt group (SHR+HS). Data represent the means ± SEM (n = 7). **: p < 0.01, *: p < 0.05.

As shown in Fig. 6A, 6B and 6C, Western blot analysis demonstrated that the high salt intake significantly increased the expressions of prorenin, renin and (P)RR of the left ventricle both in the WKYs and SHRs. The expression of (P)RR was the highest in the SHR+HS group among the groups.

### Expression of cardiac tissue angiotensinogen and angiotensin II AT1 receptors

As shown in Fig. 6D and 6E, Western blot analysis demonstrated that the high salt intake significantly increased cardiac tissue expressions of angiotensinogen and angiotensin II AT1 receptor of the left ventricle both in the WKYs and SHRs. The expressions of angiotensinogen and angiotensin II receptor were highest in the SHR+HS group among groups.

### Signal transduction

**Fig 7:**
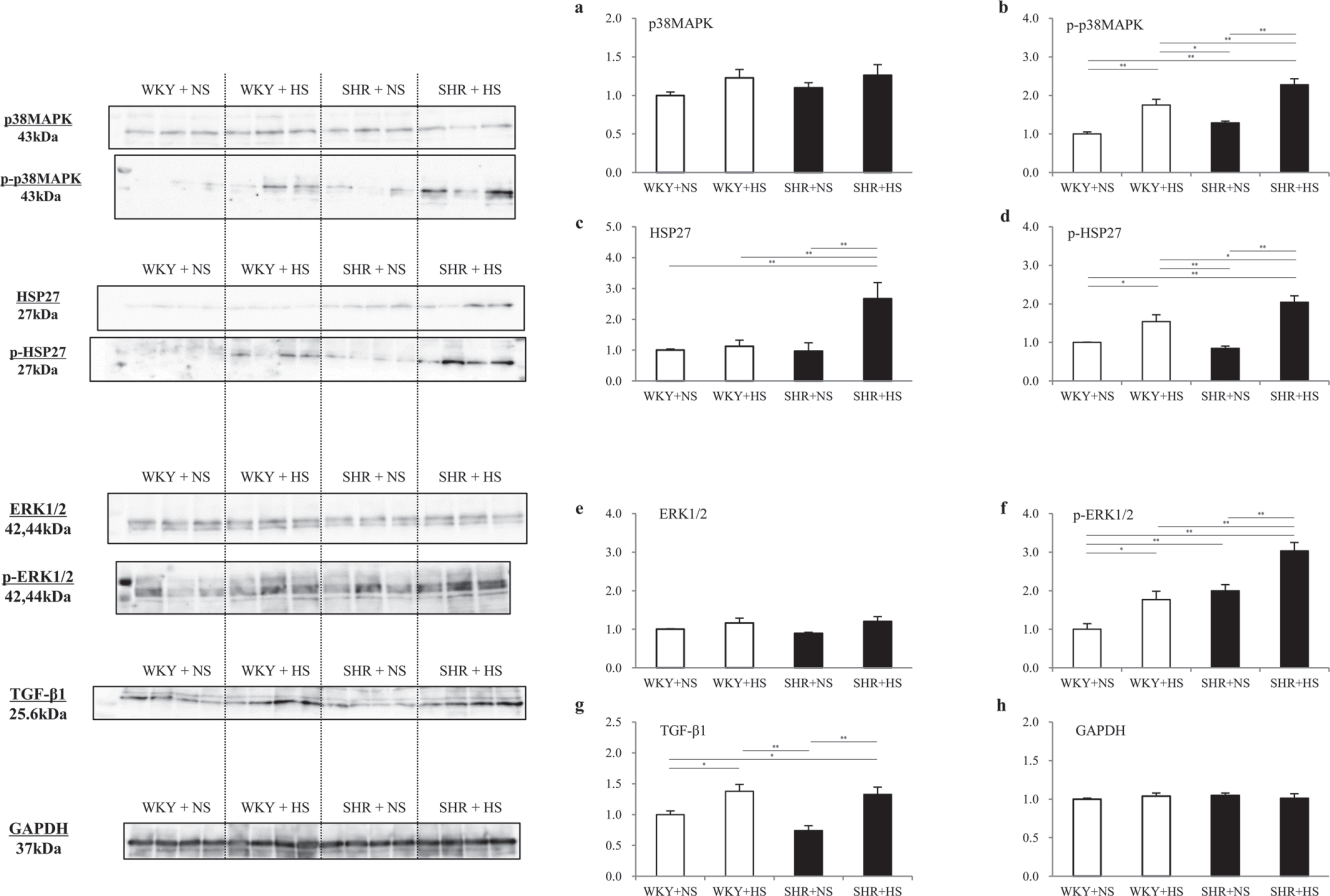
Expression of cardiac tissue p38MAPK, phosphorylated (p)-p38MAPK, HSP27, p-HSP27, ERK1/2, p-ERK1/2, TGF-β1 and GAPDH. Expressions of cardiac tissue (a) p38MAPK, (b) p-p38MAPK, (c) HSP27, (d) p-HSP27, (e) ERK1/2, (f) p-ERK1/2, (g) TGF-β1 and (h) GAPDH in the WKY + normal-salt group (WKY+NS), WKY + high-salt group (WKY+HS), SHR + normal-salt group (SHR+NS) and SHR + high-salt group (SHR+HS). Data represent the means ± SEM (n = 7). **: p < 0.01, *: p < 0.05.

Western blot analysis showed that the high salt intake significantly increased cardiac expressions of phosphorylated (p)-p38MAPK, p-HSP27, p-ERK1/2 and TGF-β1 slightly in the WKYs and substantially in the SHRs (Fig. 7B, 7D, 7F, 7G).

### Pathology

**Fig 8:**
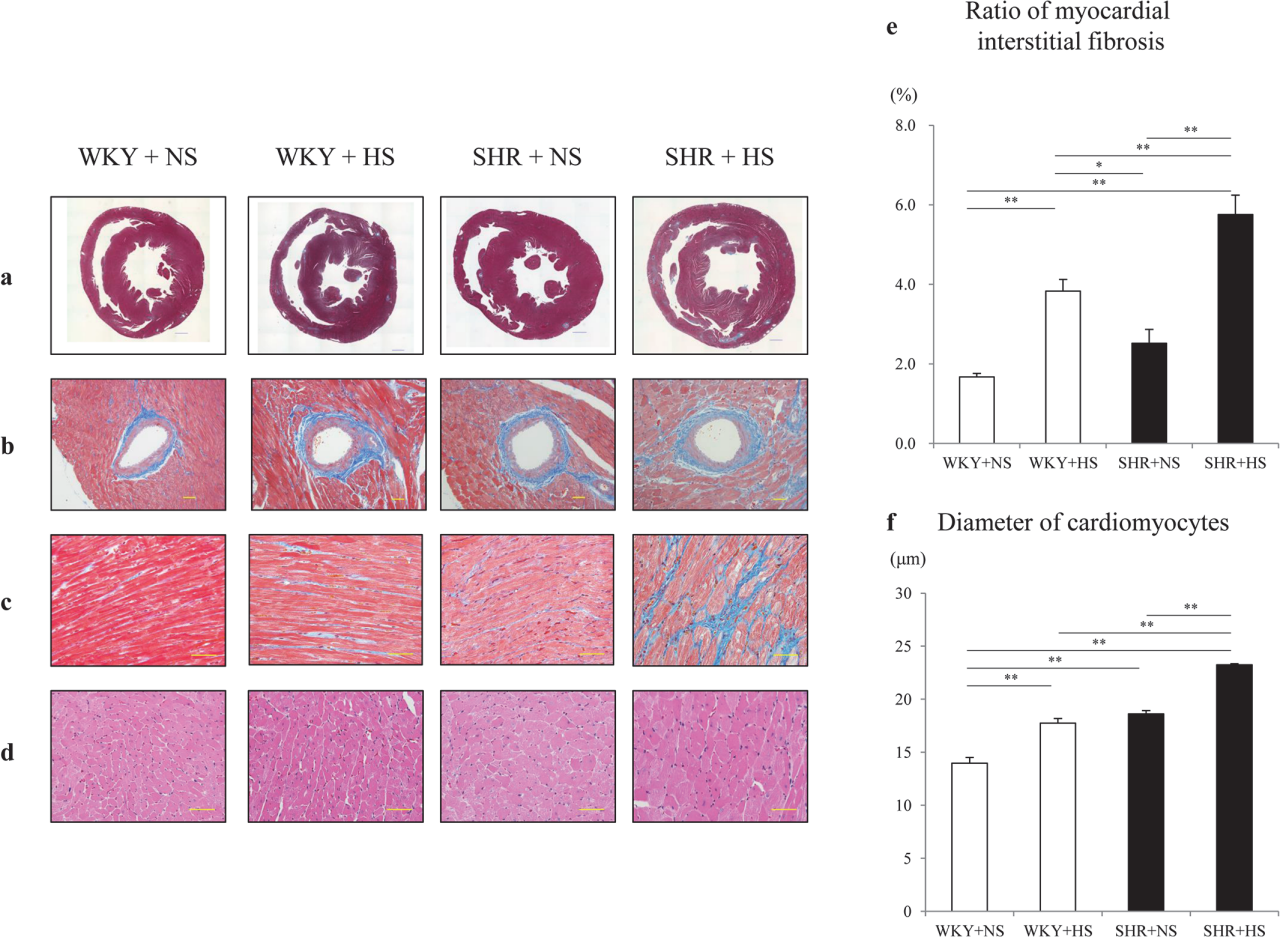
Masson-Trichrome staining and hematoxylin-eosin staining of left ventricle. Representative (a) short-axis sections of cardiac ventricle stained with Masson-Trichrome (Scale bars: 1 mm), (b) short-axis images of the myocardium and intramuscular arteries with perivascular and interstitial fibrosis stained with Masson-Trichrome (Scale bars: 50 μm), (c) short-axis images of the myocardium and interstitial fibrosis stained with Masson-Trichrome (Scale bars: 50 μm) and (d) short-axis images of the myocardium stained with hematoxylin-eosin (Scale bars: 50 μm) at 12 weeks of age in the WKY + normal-salt group (WKY+NS), WKY + high-salt group (WKY+HS), SHR + normal-salt group (SHR+NS) and SHR + high-salt group (SHR+HS). (e) Ratio of myocardial interstitial fibrosis area/ myocardium and (f) Short-axis diameter of cardiomyocytes at 12 weeks of age in the WKY + normal-salt group (WKY+NS), WKY + high-salt group (WKY+HS), SHR + normal-salt group (SHR+NS) and SHR + high-salt group (SHR+HS). Data represent the means ± SEM (n = 7). **: p < 0.01, *: p < 0.05.

Fig. 8 shows representative short axis sections of the left ventricle stained with Masson-Trichrome (Fig. 8A). The high-salt diet significantly accelerated the development of myocardial interstitial fibrosis and perivascular fibrosis slightly in the WKYs and substantially in the SHRs (Fig. 8B, 8C). The high-salt diet significantly increased the ratio of myocardial interstitial fibrosis area/ myocardium and the mean diameter of cardiomyocytes slightly in the WKYs and substantially in the SHRs (Fig. 8E, 8F) at 12 weeks of age (Ratios of myocardial interstitial fibrosis area/ myocardium were 1.67 ± 0.09, 3.83 ± 0.29, 2.52 ± 0.35 and 5.76 ± 0.49% in the WKY+NS, WKY+HS, SHR+NS and SHR+HS groups, respectively. The diameters of cardiomyocytes were 14.0 ± 0.5, 17.7 ± 0.4, 18.6 ± 0.3 and 23.2 ± 0.1 μm in the WKY+NS, WKY+HS, SHR+NS and SHR+HS groups, respectively.).

## Discussion

In the present study, the high salt intake significantly increased the systolic blood pressure both in the WKY and SHR groups as compared to those fed a normal-salt diet, as shown in Fig. 2. These results are consistent with previous reports that a high-salt diet increases the blood pressure [13, 14]. As shown in Fig. 2B, the high-salt diet gradually and significantly increased the systolic blood pressure from 6 to 12 weeks of age both in the WKY and SHR groups as compared to those fed the normal-salt diet. It has been established that the mechanism of increased blood pressure is response to high salt intake is based on the pressure natriuresis as described by Guyton [15]. It has also been reported that the mechanisms by which a high salt intake increases the blood pressure is due to increased volume load and to Na^+^/Ca^2+^ exchanger NCX1-mediated fascilitation of calcium entry into vascular smooth muscle cells and constriction [16]. In addition, it has recently been reported that neuron specific (P)RR-knockout prevents the development of salt-sensitive hypertension, suggesting that nonproteolytic activation of prorenin through binding to the (P)RR causes hypertension [17].

Among the 4 groups, the heart weight was the heaviest in the SHR+HS group, the body weight was the lightest in the SHR+HS group, and the heart weight/ body weight ratio was the highest in the SHR+HS group at 12 weeks of age. This was consistent with the previous report that high salt intake is associated with higher diet intake and lower body weight [18]. Heart failure in the SHR+HS group may be evidenced by the reduced fractional shortening and increased lung/ body weight ratio, which means pulmonary congestion induced by a combination of hypertension and a high salt intake.

Left ventricular hypertrophy assessed by IVS thickness with echocardiography was greater in the WKY+HS group than in the WKY+NS, and greater in the SHR+HS group than in the SHR+NS group, as shown in Fig. 4. This may be at least in part due to a higher systolic blood pressure because the high salt intake significantly increased the systolic blood pressure both in the WKYs and SHRs. However, there has been a report demonstrating that myocardial hypertrophy due to high salt intake is blood pressure independent [11, 12]. In the present study, therefore, the activation of p38MAPK and HSP27 may have played an important role in the increased myocyte size and IVS thickness both in the WKY+HS and SHR+HS groups. Fractional shortening (FS) was significantly smaller in the SHR+HS group as compared to the other groups. Impaired FS may have been mechanically caused by an increase in the systolic blood pressure, an afterload to the left ventricle, and also by myocardial hypertrophy caused by the high salt intake [11] in the SHR group.

It has generally been accepted that PRA is affected by salt intake. It has been reported that a high salt intake causes a reduction in PRA [7] and a low salt intake causes a rise in PRA [8]. In the present study, consistent with previous reports [7, 8], the high-salt diet significantly decreased PRA or plasma angiotensin II both in the WKY and SHR groups, as shown in Fig. 5.

It has been reported that the tissue level of the renin angiotensin system behaves independently of PRA, especially in diabetes; the tissue renin-angiotensin system is activated even though PRA is low in diabetes [9, 10]. In the present study, the expressions of prorenin, renin and (P)RR were significantly enhanced in the left ventricle by high salt loading both in the WKY and SHR groups (Fig. 6), although PRA was low. This suggests that the cardiac tissue RA system is activated by a high salt intake through activation of the cardiac (pro)renin receptor both in the WKY and SHR groups. Prorenin and renin bound to (P)RR have been reported to trigger intracellular signaling and the activation of extracellular signal-related kinases (ERK)1/2, leading to the upregulation of TGF-β1, which induces fibrosis [19, 20], and it has been reported that the activation of (P)RR contributes to the development of cardiac fibrosis in genetic hypertension [21]. In addition, stimulation of (P)RR has been reported to trigger the activation of p38MAPK, leading to the upregulation of HSP27, which enhances the synthesis of DNA and induces cardiomyocyte hypertrophy [22, 23]. In the present study, we observed the significant activation of ERK1/2 and TGF-β1 (Fig. 7F, 7G) as well as significant activation of p38MAPK and HSP27 (Fig. 7B, 7D) in cardiac tissues, by high salt intake, leading to cardiac interstitial fibrosis, perivascular fibrosis and cardiomyocyte hypertrophy slightly in the WKY groups and substantially in the SHR group.

In the present study, the high salt intake significantly enhanced myocardial expression of angiotensinogen and angiotensin II AT1 receptor slightly in the WKYs and substantially in the SHRs (Fig. 6D, 6E), suggesting that the high salt intake enhances myocardial expression of angiotensinogen and angiotensin II AT1 receptor. It was recently reported that the fibrotic response to angiotensin II is mediated by the angiotensin II AT-1 receptor and requires p38MAPK phosphorylation and a subsequent increase in the expression of TGF-β1, but does not require ERK 1/2 phosphorylation in skeletal muscle cells [24]. Therefore, downstream signals of angiotensin II AT1 receptor have been suggested to be p38MAPK and TGF-β1. In the present study, the phosphorylation of p38MAPK was enhanced and TGF-β1 was activated by high salt intake slightly in the WKYs and substantially in the SHRs. This suggests that the combination of hypertension and a high salt intake is required to enhance the myocardial expression of angiotensinogen and angiotensin II AT1 receptor, and thus, substantial the activation of p38MAPK and TGF-β1.

Pathologically, in the present study, cardiac interstitial fibrosis, perivascular fibrosis and cardiomyocyte hypertrophy were accelerated slightly in the WKY+HS group and substantially in the SHR+HS group (Fig. 8), suggesting that the combination of hypertension and a high salt intake substantially accelerates cardiac interstitial fibrosis, perivascular fibrosis and cardiomyocyte hypertrophy both in the WKYs and SHRs.

Prorenin is inactive because it contains a prosegment which covers the enzymatic cleft and inhibits the access of angiotensinogen. However, on binding to the (P)RR, prorenin becomes enzymatically active by uncovering of the prosegment from the enzymatic cleft through nonproteolytic activation [25]. Then, binding of prorenin to (P)RR not only triggers intracellular signaling by stimulating (P)RR but also stimulates angiotensin II AT1 receptor by angiotensin II formation through promoting the access of angiotensinogen.

**Fig 9:**
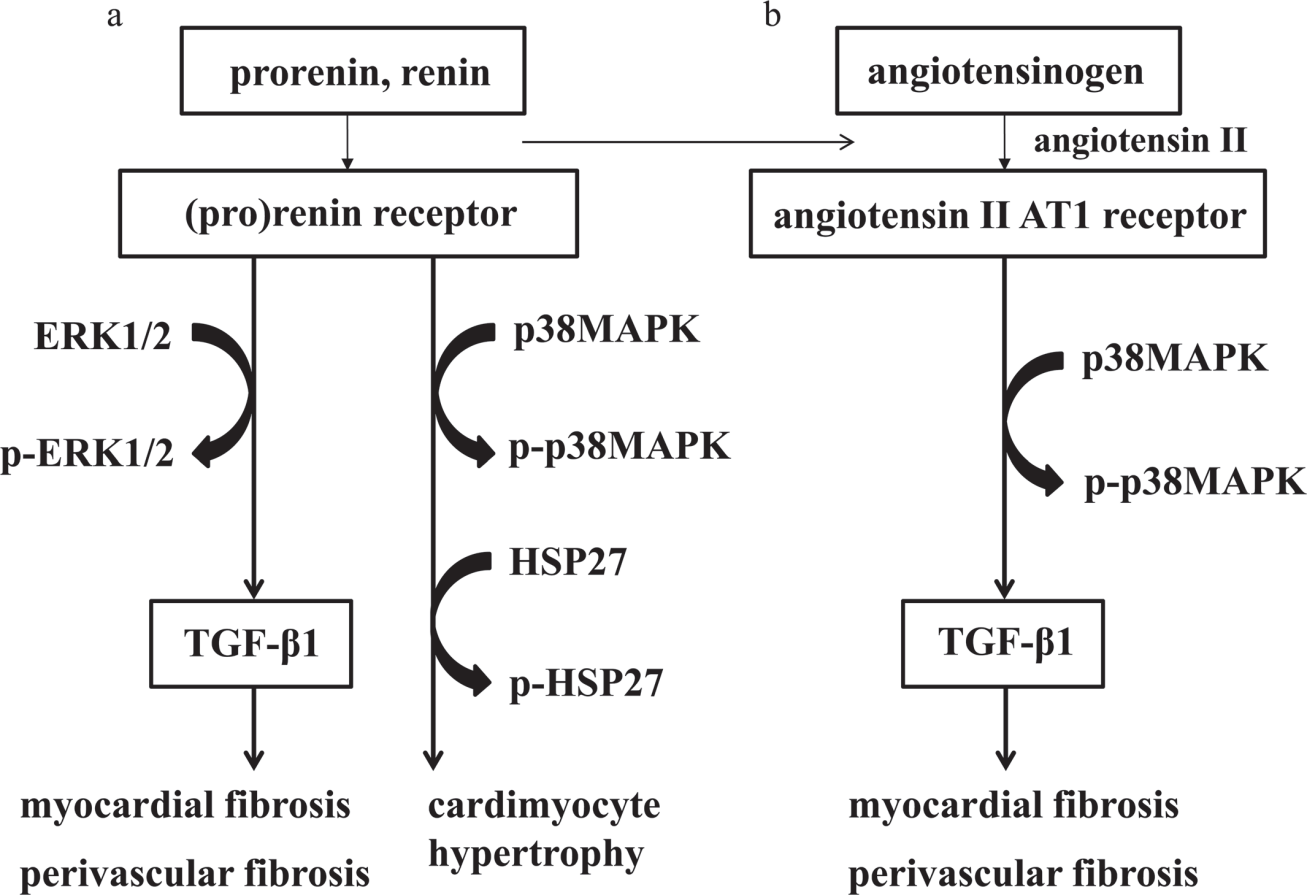
The proposed mechanism by which the combination of hypertension and a high salt intake causes myocardial fibrosis, myocardial perivascular fibrosis and cardiomyocyte hypertrophy, as noted in the present study. (a) signal transduction through (pro)renin receptors. (b) signal transduction through angiotensin II AT1 receptors. Both a and b contribute to the effects of the combination of hypertension and a high salt intake.

Fig. 9 shows the proposed mechanism by which the combination of hypertension and a high salt intake produces myocardial fibrosis, myocardial perivascular fibrosis and cardiomyocyte hypertrophy, as noted in the present study. Fig. 9A shows signal transduction through (pro)renin receptor, and Fig. 9B shows that through angiotensin II AT1 receptors, both of which contribute to the effects of the combination of hypertension and a high salt intake.

However, the precise mechanism by which a high salt intake accelerates the upregulation of myocardial (P)RR and activates its downstream signal transduction in hypertension remains to be investigated. Further investigation is warranted.

## Conclusions

The high salt intake enhanced cardiac expressions of prorenin, renin and (pro)renin receptor as well as the upregulation of cardiac angiotensinogen and angiotensin II AT1 receptor, and activated its downstream signals ERK1/2, TGF-β1, p38MAPK and HSP27, leading to the acceleration of cardiac interstitial fibrosis, perivascular fibrosis and cardiomyocyte hypertrophy at an early stage of hypertension.
